# Utility of bronchoalveolar lavage for COVID-19: a perspective from the Dragon consortium

**DOI:** 10.3389/fmed.2024.1259570

**Published:** 2024-02-02

**Authors:** Sara Tomassetti, Luca Ciani, Valentina Luzzi, Leonardo Gori, Marco Trigiani, Leonardo Giuntoli, Federico Lavorini, Venerino Poletti, Claudia Ravaglia, Alfons Torrego, Fabien Maldonado, Robert Lentz, Francesco Annunziato, Laura Maggi, Gian Maria Rossolini, Simona Pollini, Ombretta Para, Greta Ciurleo, Alessandro Casini, Laura Rasero, Alessandro Bartoloni, Michele Spinicci, Mohammed Munavvar, Stefano Gasparini, Camilla Comin, Marco Matucci Cerinic, Anna Peired, Monique Henket, Benoit Ernst, Renaud Louis, Jean-louis Corhay, Cosimo Nardi, Julien Guiot

**Affiliations:** ^1^Interventional Pulmonology Unit, Department of Experimental and Clinical Medicine, Careggi University Hospital, Florence, Italy; ^2^Pulmonology Unit, Department of Experimental and Clinical Medicine, Careggi University Hospital, Florence, Italy; ^3^Department of Diseases of the Thorax, GB Morgagni Hospital, Forlì, Italy; ^4^Respiratory Department, Hospital de la Santa Creu i Sant Pau, Barcelona, Spain; ^5^Division of Allergy, Pulmonary and Critical Care Medicine, Department of Thoracic Surgery, Vanderbilt University Medical Center, Nashville, TN, United States; ^6^Department of Experimental and Clinical Medicine, University of Florence, Florence, Italy; ^7^Department of Experimental Medicine, University of Florence, Florence, Italy; ^8^Microbiology and Virology Unit, Florence Careggi University Hospital, Florence, Italy; ^9^Internal Medicine Unit 1, AOU Careggi, Florence, Italy; ^10^Internal Medicine Unit 2, AOU Careggi, Florence, Italy; ^11^Department of Health Science, Clinical Innovations and Research Unit, Careggi University Hospital, Florence, Italy; ^12^Infectious and Tropical Diseases Unit, Department of Experimental and Clinical Medicine, University of Florence, Florence, Italy; ^13^School of Biological Sciences, The University of Manchester, Manchester, United Kingdom; ^14^Department of Respiratory, Lancashire Teaching Hospital NHS Foundation Trust, Preston, United Kingdom; ^15^Interventional Pulmonology Unit, University Hospital Riuniti di Ancona, Ancona, Italy; ^16^Department of Experimental and Clinical Medicine Section of Surgery, Histopathology, and Molecular Pathology, University of Florence, Florence, Italy; ^17^Department of Clinical and Experimental Biomedical Sciences, University of Florence, Florence, Italy; ^18^Department of Respiratory Medicine, Universitary Hospital of Liège, Liège, Belgium; ^19^Department of Experimental and Clinical Biomedical Sciences, Radiodiagnostic Unit n. 2, University of Florence, Florence, Italy

**Keywords:** COVID-19, bronchoalveolar lavage, interstitial pneumonia, infections, interventional pulmonology

## Abstract

Diagnosing COVID-19 and treating its complications remains a challenge. This review reflects the perspective of some of the Dragon (IMI 2-call 21, #101005122) research consortium collaborators on the utility of bronchoalveolar lavage (BAL) in COVID-19. BAL has been proposed as a potentially useful diagnostic tool to increase COVID-19 diagnosis sensitivity. In both critically ill and non-critically ill COVID-19 patients, BAL has a relevant role in detecting other infections or supporting alternative diagnoses and can change management decisions in up to two-thirds of patients. BAL is used to guide steroid and immunosuppressive treatment and to narrow or discontinue antibiotic treatment, reducing the use of unnecessary broad antibiotics. Moreover, cellular analysis and novel multi-omics techniques on BAL are of critical importance for understanding the microenvironment and interaction between epithelial cells and immunity, revealing novel potential prognostic and therapeutic targets. The BAL technique has been described as safe for both patients and healthcare workers in more than a thousand procedures reported to date in the literature. Based on these preliminary studies, we recognize that BAL is a feasible procedure in COVID-19 known or suspected cases, useful to properly guide patient management, and has great potential for research.

## Introduction

The rapid outbreak of coronavirus disease 2019 (COVID-19), related to severe acute respiratory syndrome coronavirus 2 (SARS-CoV-2) infection, has been a public health emergency of international concern. Diagnosing COVID-19, treating its complications, and predicting how the disease will progress in different patients remains a challenge. The DRAGON project (IMI 2-call 21, #101005122) draws on new and existing data and sample collection efforts to carry out a detailed profiling of patients. In the Dragon consortium, Florence University, Italy, and Centre Hospitalier Universitarie de Liege, Belgium, have focused their research on the role of interventional pulmonology (IP) and bronchoalveolar lavage (BAL) sample collection in COVID-19. This document reflects the perspective of some of the Dragon research consortium collaborators on the utility of BAL in COVID-19.

Interventional pulmonology in patients with COVID-19 is required to manage complications (atelectasis, hemoptysis, pneumothorax, and pleural effusions) and guide airway management (airway secretion management, intubation, or tracheostomy guide). BAL in COVID-19 has been used to obtain samples for both cytology and microbiology purposes (detecting infections and differential diagnosis with other interstitial lung disorders). If the role of IP in treating COVID-19 complications and guiding airway management is well-established, the role of BAL in COVID-19 diagnosis and management has been questioned. Bronchoscopy is an aerosol-generating procedure, and its routine use in COVID-19 patients has been discouraged (1). However, avoiding bronchoscopy in COVID-19 patients exposes physicians to risks of misdiagnosis and suboptimal treatment. BAL is a well-established minimally invasive technique that has an important diagnostic role and has been routinely used for decades for the diagnosis of infectious, neoplastic, and non-neoplastic diffuse lung diseases. BAL clinical role in the diagnosis of respiratory infection is of utmost importance (2–5). Therefore, BAL has been used in many expert centers to manage COVID-19 and in several research protocols to investigate COVID-19 pathogenetic mechanisms.

We aimed to review the current evidence supporting the role of BAL in the diagnosis of COVID-19 infection, in the detection of coexisting infections, and in understanding COVID-19 features and pathogenetic mechanisms.

## Limits of the current diagnostic approach for COVID-19

The diagnostic gold standard for COVID-19 is the naso-pharyngeal (NP) swab reverse-transcription real-time polymerase chain reaction (rRT-PCR) detection of SARS-CoV-2. However, the lack of a shared reference standard for COVID-19 diagnosis prevents reliable data on the sensitivity of NP swabs. Clerici et al. assessed nasopharyngeal swab sensitivity in patients with known SARS-CoV-2 infection based on the presence of symptoms and of ≥1 positive rRT-PCR serial testing and found a sensitivity of 77% (95%CI, 73–81%) (6). Wang et al. evaluated SARS-CoV-2 detectability in different biological specimens in COVID-19 patients and found an NP swab sensitivity of 63% (7). Pooled data found that the probability of a false negative result was as high as 21% even at the optimal testing window (3 days after symptom onset) (8).

Given the limits of NP swab testing, some experts propose to diagnose suspected cases using the widely available, time-saving, and non-invasive imaging approach of chest computed tomography (CT), which could serve as an efficient and effective way to flag, diagnose, and possibly triage COVID-19 patients (9, 10). However, as confirmed by a metanalysis of 60 studies (5,744 patients), CT has a low specificity compared to NP swabs rRT-PCR, 46% (95%CI, 29–63%) (11). Ongoing studies are evaluating the role of radiomics analysis to identify a diagnostic signature for COVID-19 infection, based on standard-of-care chest CT imaging, with promising preliminary results showing a sensitivity of 69.52% and a specificity of 91.63% (12).

In this scenario, identifying the false negative cases remains of critical importance to properly manage patients, avoiding improper allocation of COVID-19 cases and allowing a timely treatment. Since the early pandemic, BAL has been indicated as a potentially useful diagnostic tool to increase COVID-19 diagnosis sensitivity. Nevertheless, considering the high potential for aerosol exposure generated during BAL, international bronchology societies have universally cautioned about the limited and proper use of this tool in clinical practice during the pandemic peaks. The role of BAL in the diagnostic algorithm of COVID-19 has been debated and explored in several studies.

## Indications of major bronchoscopy societies

Several bronchology societies have issued documents regarding bronchoscopy during the early phase of the COVID-19 pandemic (1, 13–16). Based on the risk of aerosol-transmitted infection, all societies at that time recommended postponing elective procedures, limiting the number of procedures in COVID-19 patients, performing procedures in COVID-19 patients with minimal sufficient staff, and with the use of appropriate personal protective equipment (PPE). Deciding how to stratify elective procedures to minimize the risk of transmission while not compromising time-sensitive medical care has been a major challenge, and experts recommended reviewing the need for all procedures on a case-by-case basis to assess the indication and urgency (14).

Known or suspected COVID-19 infection was considered a relative contraindication to bronchoscopy, given the uncertain benefits and possible risks. Bronchoscopy in COVID-19 patients had three main roles: (1) the diagnosis of SARS-CoV-2 infection when other diagnostic tools were inconclusive; (2) the identification of co-infections or superinfections in patients with worsening respiratory conditions; and (3) the treatment of bronchoscopic emergencies (massive bleeding, significant airway stenosis, and airway secretions causing tracheobronchial obstruction).

The major bronchoscopy societies agreed on the need to limit the use of BAL in the diagnosis of SARS-CoV-2 infection. However, based on the need to avoid false negatives, the societies made a point for a possible indication to perform BAL in cases of suspected COVID-19 when other diagnostic methods were inconclusive and in those situations in which the identification of coinfections could play an important role in the therapeutic decision.

None of these bronchology society’s indications given during the early pandemic phase were comprehensive, and significant uncertainty remained regarding whom to perform bronchoscopy (17). At that time, no data specific to bronchoscopy in COVID-19 were yet available, and the recommendations were experts’ opinions derived from observations made during prior respiratory viral outbreaks, including other SARS, Middle East Respiratory Syndrome, and influenza. However, in the rapidly changing clinical environment of the last 2 years, many centers equipped with appropriate PPE and experienced in the use of BAL have performed BAL in known or suspected COVID-19 infections, generating new evidence on the utility of bronchoscopy in COVID-19 that needs to be carefully considered.

## BAL in suspected COVID-19 non-critically ill patients

As recently reported by systematic reviews and metanalysis, several retrospective and few prospective observational studies have investigated the role of BAL in suspected or known COVID-19 (18, 19). To the best of our knowledge, all studies performed on non-critically ill patients are retrospective. A summary of BAL findings in non-critically ill patients is reported in Tables 1, 2. Between January and February 2020, Chinese scientists reported 5 cases of suspected COVID-19, with BAL showing positivity for SARS-CoV-2 in all cases (20). Subsequently, between March and May 2020, Italian virologists confirmed a higher positivity in BAL compared to other specimens (15%, 55/367 positive BAL, compared to 8%, 769/9461 positive NP swabs) (21). A small retrospective case series reported a 19% prevalence (3/19 cases) of SARS-CoV-2 infection in BAL performed in patients with negative NP swabs (22). During the first COVID-19 wave (March–April 2020), De Clercq et al. conducted a retrospective monocenter study in Belgium to evaluate the feasibility of their local diagnostic protocol that included BAL in patients’ diagnostic workup (23). They performed 27 BAL in non-critically ill patients with HCRT changes suspected for COVID-19 and two negative NP swabs and found 26% (7/27) positive BAL for SARS-CoV-2. They also identified one coinfection in SARS-CoV-2 positive (*E. cloacae*) and 63% of other pathogens in negative BAL for SARS-CoV-2, including *Mycoplasma pneumoniae, Streptococcus pneumoniae, Haemophilus influenzae, Pneumocysitis jirovecii*, and other viruses (23). Another retrospective study conducted in two Belgian centers during the first wave confirmed the utility of BAL in detecting SARS-CoV-2 in 25% (14/55) of non-critically ill patients with negative NP swabs (24). The authors also underlined the utility of BAL in therapeutic management that was changed after BAL in 60% of cases (33/55), either because other pathogens were identified (one coinfection with *Serratia marcescens* in SARS-CoV-2 positive cases and 42%, 23/55 of other pathogens in SARS-CoV-2 negative cases, including *Mycobacterium tuberculosis, Pneumocystis jirovecii, Haemophilus, Serratia, Escherichia coli, virus Influenza type A, Metapneumoviruses, Herpes viruses, and Aspergillus fumigatus*) or because an alternative diagnosis was made (18% of cases, 10/55, including rheumatoid arthritis, hypersensitivity pneumonitis, cardiogenic edema, cryptogenic organizing pneumonia, and hepatopulmonary syndrome), allowing appropriate immunosuppression (24). During the first COVID-19 wave, Mondoni et al. carried out in Italy an observational, retrospective, multicenter cohort study aimed to evaluate the diagnostic yield of bronchoscopy in patients with two negative NP swabs and suspected COVID-19 (25). A total of 109 adults, 71% males and of age 60 (SD 13.6) years, were enrolled, and 108 bronchoscopies (99%) were performed with the flexible scope and 13 with rigid. Two-thirds of the procedures (*N* = 78) were performed to confirm a COVID-19 diagnosis, and one-third were urgent/life-saving procedures. Only 10% of the procedures were carried out in the ICU setting (8.2% invasive ventilation, 1.8% ECMO). The diagnostic yield of bronchoscopy to detect SARS-CoV-2 in patients with previous negative swabs and clinical and radiological suspicion of COVID-19 pneumonia was 55.1% (43/78); 1.8% (2/109) of patients with both NP swabs and BAL negative for SARS-CoV-2 showed a late NP swab positivity. Coinfections were detected in 4 cases (3.6% of the total): *Haemophilus influenzae, Aspergillus fumigatus, Aspergillus* spp.*, and Candida albicans* (25). In the same period (March–April 2020), Patrucco and coworkers conducted a similar Italian observational, retrospective, multicenter cohort study, including 131 suspected COVID-19 with two negative NP swabs (male 71%, age 65 years, range 54–74 years), the majority in Internal Medicine ward (63%), 27.5% in sub-intensive unit, and 9% in ICU. SARS-CoV-2 was isolated in 43 (32.8%) BAL (26). Positive patients were younger compared to the negative ones (56 vs. 67, *p* = 0.004) and showed a higher HRCT involvement (ground-glass, peripheral, posterior, and multilobar involvement) (26). Other microbiological findings were identified in 26 cases (19.8%) and included *Herpesviruses, Cytomegalovirus, Staphylococcus aureus, Escherichia coli, Klebsiella pneumoniae, Pseudomonas aeruginosa, and fungi*. Considering both the identification of COVID-19 and the detection of other causal agents, BAL microbiological analysis was considered clinically useful in 67% of cases (26). Barberi et al., in a population of hospitalized patients for suspected COVID-19, negative NP swabs, and mild–moderate disease severity (PaO2/FiO2 307, range 254–362), confirmed a BAL positivity of 16% (32/198) and 9% (5/54 in patients with negative HRCT) (27). Moreover, BAL detected 12.5% (4/32) of coinfections in SARS-CoV-2 positive patients and 33% other infections in SARS-CoV-2 negative patients. The logistic regression analysis detected two factors predictive of BAL positivity: fever (OR 1.94 per additional °C, 95%CI 1.13–3.33, *p* = 0.016) and HRCT scan involvement grade 2 or more (OR 7.36, 95%CI 2.10–25.77, *p* = 0.002) (27). In contrast to those results, three Italian single-center observational retrospective studies on BAL conducted in the same time period (March–May 2020) in suspected COVID-19 with negative NP swabs (*N* = 81, *N* = 79 and *N* = 28 patients, respectively) showed poor BAL performance in detecting SARS-CoV-2 infection with 3/81 (3.7%), 2/79 (2.5%), and 0/28 positive BAL (28–30). In those studies, BAL negative for SARS-CoV-2 was still useful for identifying other microorganisms (*mycobacteria, Pneumocystis, Haemophilus parainfluenzae, Staphylococcus, Pseudomonas, Streptococcus, Enterobacterales, Klebsiella, Candida, and other viruses*) (29, 30). Two American studies found a 100% concordance between negative NP swabs and BAL conducted in patients who were screened for SARS-CoV-2 before an elective bronchoscopy for suspected diseases other than COVID-19 (obstructive diseases, interstitial lung disease, and lung transplant surveillance) (31, 32). In the study conducted by Oberg et al., all but one patient had HRCT non-suggestive for COVID-19 (negative HRTC in 58% and indeterminate or atypical in the remaining cases), and none had clinical-laboratory features of COVID-19 (31). This study suggests that when the clinical-radiological scenario is not suggestive of COVID-19 and the NP swabs is negative, BAL for COVID-19 is unlikely to be useful, even during a pandemic peak.

**Table 1:** Summary of BAL findings in critically and non-critically ill COVID-19 patients.

	Critically ill COVID-19	Non-critically COVID-19
% of SARS-CoV-2 positive BAL in negative NP swab (ref)	3–18% (ref 38, 39)	0–55% (ref 22–30)
% of coinfections detected by BAL in COVID-19 patients	21–54% (ref 33, 38, 40)	2–37% (ref 23–26)
% of infections detected by BAL in negative SARS-CoV-2	16–54% (ref 38, 39)	19–63% (ref 22–26)
% of diagnoses of non-infectious diseases in which BAL was helpful	N/A	18% (ref 24)
Overall % of cases in which BAL was considered clinically helpful	44–71% (ref 33, 40)	60–67% (ref 23, 25)

**Table 2:** Reported infections in BAL of critically and non-critically ill patients, with positive or negative SARS-CoV-2 BAL findings.

	Critically ill		Non critically ill	
BAL SARS-CoV-2	Positive	Negative	Positive	Negative
Bacterial infections	*Enterobacteriacee* (*Escherichia coli*, *Klebsiella pneumoniae*, *K. aerogenes*, *E. cloacae*, *E. fecalis*, *K. aerogenes*)			
	*Pseudomonas*			
	*Stenotrophomonas maltophilia*	*Staphylococcus*	*Haemophilus influenzae e parainfluenzae*	
	MRSA		*Serratia marcescens*	
			*Streptococcus pneumoniae*	
			*Staphylococcus aureus*	
				*Mycoplasma pneumoniae*
Mycobacterial infections		*Mycobacterium avium*		*Mycobacterium tuberculosis*
Fungal infections	*Pneumocystis jirovecii*			*Pneumocystis jirovecii*
			*Aspergillus fumigatus*	
			*Candida*	
Viral infections			*Cytomegalovirus*	Influenza A
				*Metapneumoviruses*
				*Herpes viruses*

Methicillin-resistant *S. aureus* (MRSA).

Among these small retrospective studies, there is a notable variation in the reported utility of BAL for detecting SARS-CoV-2. This suggests that several factors may influence BAL diagnostic accuracy in detecting SARS-CoV-2, including the heterogeneity of the populations, the variability in BAL technique, and sample processing. Moreover, different treatment strategies and BAL timing can impact the results. Concomitant broad-spectrum antibiotic and antiviral therapies may reduce the effectiveness of BAL in these patients. It is important to mention that BAL diagnostic yield for COVID-19 detection is also influenced by the epidemiological incidence of the disease and may be influenced by the viral variant. With the changing epidemiological scenario and novel variants, the BAL diagnostic yield could significantly change. Prospective studies conducted in larger and more recent populations are needed, particularly considering that the clinical scenario is rapidly changing due to the emergence of novel variants in the vaccinated population.

## BAL in COVID-19 in critically ill patients

Several studies have evaluated the utility of BAL in critically ill patients, and three were prospective (18, 19, 33–36). A summary of BAL findings in critically ill patients is reported in Tables 1, 2. The highest positivity for SARS-CoV-2 detection in BAL performed in critically ill patients has been reported by Wang et al., 93% (95%CI 074–1.00; *N* total BAL = 15), and Yang et al., 68% (95%CI 056–0.79; *N* total BAL = 44) (7, 19, 37). The latter study reported a 100% SARS-CoV-2 positivity in more severe patients in whom BAL was collected within the first 2 weeks. After 15 days, the positivity of nasopharyngeal and oropharyngeal swabs decreased, while BAL maintained a high positive rate of 63% (37). Gao et al. designed a retrospective study to evaluate the diagnostic accuracy of the nasopharyngeal swab (NP) compared to BAL for the detection of SARS-CoV-2 (38). They reviewed 123 intubated patients who underwent both tests (time interval median 1 day, IQR 1–2.75 days), showing that 9 cases with negative NP swabs had positive BAL, 7% of the total. The remaining cases were as follows: 70 positives for both, 39 negatives for both, and 5 cases with positive NP swabs and negative BAL. Bacterial pneumonia was identified in 34% of total cases, with significantly more bacterial coinfections in the non-COVID-19 (24/44, 54%) than in the COVID-19 patients (18/79, 23%) (38). Similar results were achieved by Mahmood et al. in 55 critically ill patients; in the subgroup of 37 negative NP swabs, they found one positive BAL for SARS-CoV-2 (3%), and in the overall cohort, they found 16% of positive cultures other than COVID-19 (*Staphylococcus, Pseudomonas, Fungi, Mycobacterium avium, and Pneumocysist jirovecii*) (39). In the ICU setting, BAL allows the detection of coinfections in a significant proportion of COVID-19 (Table 2). In several studies conducted in the ICU setting, BAL was mainly performed for a microbiological purpose, with a significant impact on subsequent medical decisions. Baron et al. performed 28 BAL in 24 patients for microbiological purposes. Only in 2 (7%) patients were BAL performed to confirm COVID-19 after a negative NP swab (40). The authors describe the use of BAL mainly for suspicion of ventilator-associated pneumonia (*N* = 11, 39%) and invasive aspergillosis (*N* = 4, 14%) and to rule out superinfection before starting a steroid course. In this study, BAL had an impact on medical decisions in 20 cases out of 28 (71%), with introduction (*n* = 6), continuation (*n* = 3), switch (*n* = 2), or withdrawal (*n* = 4) of antimicrobial therapy in 14 cases (50%) and/or decision to start (*n* = 6; 21%), or not (*n* = 6, 21%), corticosteroid therapy (40). Pickens et al. conducted a retrospective single-center study in COVID-19 mechanically ventilated patients, documenting by early BAL (48 h within intubation) 21% (28/133) of bacterial superinfection pneumonia. Streptococcus species and methicillin-susceptible *S. aureus* (MSSA) combined accounted for 79% (22/28) of cases (33). Polymicrobial infections were common; three patients, previously treated with antibiotics, had pathogens resistant to standard CAP antibiotics—one *Stenotrophomonas maltophilia* and two methicillin-resistant *S. aureus* (MRSA)—and Pneumocystis was found in one patient with HIV on antiretroviral treatment. For each day of mechanical ventilation, they measured the Narrow Antibiotic Treatment (NAT) score and found a clinically and statistically significant difference between positive and negative BAL results (NAT score median difference − 1, 95%CI −1 to 0; *p* = 0.001). Bacterial ventilator-associated pneumonia developed in 44% of patients and could not be accurately identified in the absence of microbiologic analysis of BAL fluid. In a recent prospective single-center trial, 79 COVID-19 ventilated patients were tested with BAL, BAL_FAPPP_ (fast microbiology FILMARRAY Pneumonia Panel plus), and endotracheal aspirate (ETA). Positive microbiology was detected in 34% (27/79) of BAL and 44% (35/79) of ETA. The incidence rate of microbiologically confirmed VAP was 33.1 (95%CI 22.1–44.0) and 20.1 (95%CI 12.5–27.7), according to ETA and BAL, respectively. With BAL as the reference standard, ETA showed 88.9% (95%CI 70.8–97.7) sensitivity and 50.0% (95%CI 28.2–71.8) specificity (Cohen’s Kappa 0.40, 95%CI 0.16–0.65). BAL_FAPP_ showed 95.0% (95%CI 75.1–99.9) sensitivity and 69% (95%CI 49.2–84.7) specificity (Cohen’s Kappa 0.60, 95%CI 0.39–0.81). These findings show that heterogeneity in microbial findings depends on the respiratory sampling (ETA vs. BAL) and the diagnostic technique employed (molecular microbiology vs. conventional culture). The specificity of other sampling methods is limited, and the concordance with BAL is low (41).

These findings suggest that negative BAL analysis was used to narrow or discontinue antibiotic treatment and that in the absence of a BAL, ventilator-associated pneumonia may be underrecognized yet overtreated with unnecessary broad antibiotics (33, 41).

## BAL utility for post-COVID ILD assessment

Emerging data suggest that after hospitalization for COVID-19, approximately half of the survivors develop inflammatory pulmonary sequelae and one-third of cases present with pulmonary fibrosis (42). Inflammatory changes reduce over time, whereas fibrotic changes tend to be more persistent (42). Parenchymal bands do not impact lung function and do not represent a clinically significant finding, whereas the features of fibrotic changes of irreversible fibrosis (reticulation, traction bronchiectasis or bronchiolectasis, and pulmonary distortion) do, but remain stable over a 12-month period (43). The diagnostic and prognostic role of BAL and lung biopsy in post-COVID-ILD has been poorly investigated. Combining the clinical, radiological, pathological, and BAL features of 164 post-COVID cases, Ravaglia et al. recently described three different phenotypes of post-COVID-ILD: (1) prominent vascular changes, (2) acute/subacute injury, and (3) pre-existing chronic fibrosing ILD (44). The authors showed that the morphological abnormalities seen in histopathologic samples from transbronchial cryobiopsies were mirrored by BAL features. In cluster 2, lymphocytosis (>20%) was associated with organizing pneumonia (OP) and abundant lymphoid interstitial and perivascular infiltrate. In cluster 3, BAL combined with other features can help in defining the type of ILD and may add useful information to discriminate post-COVID ILD from other IIPs (e.g., hypersensitivity pneumonitis). All BAL described by Ravaglia et al. were negative for SARS-CoV-2, confirming that the pulmonary changes are related to pathogenic mechanisms that perpetuate after viral clearance (44). However, it has been described that immunocompromised patients (hematologic malignancies on active treatment) can present with virus persistence in BAL (45). These cases respond to antiviral treatment with no need for corticosteroids, which remain the main treatment for cases with OP but without persistent infection. In cases of persistent infection, BAL shows lymphocytosis and histopathology cellular NSIP/OP pattern, indistinguishable from cluster 2 described by Ravaglia et al. (unpublished observation from S Tomassetti). Therefore, when NS is negative, BAL becomes the only diagnostic test that allows the detection and appropriate treatment of persistent SARS-CoV-2 infection. The presence of neutrophilia on BAL can correlate with fibrotic changes (normal BAL or mild neutrophilia) or with underlying coinfections (more prominent neutrophilia) that can also be detected by BAL microbiological analysis.

## Comparison among BAL, mini-BAL, and bronchial wash

Currently, there are no studies designed to compare the diagnostic yield and complications of BAL, mini-BAL, and bronchial wash. BAL consists of the instillation of approximately 120 mL of saline solution with the flexible scope wedged into a segmental bronchus. This technique allows the collection of the distal (broncho-alveolar) cellular and acellular components of the lung. The instillation of at least 100 mL of saline solution is required to reach the alveolar component and achieve a BAL of sufficient quality for microbiology, cytology, immunological, and molecular studies (46). For patients with severe respiratory failure or poor general conditions, bronchial wash or mini-BAL are possible alternative methods for microbiology studies. Bronchial wash collects the bronchial component and is performed with approximately 20 mL of saline solutions within the main or lobar bronchi. This technique does not allow the study of the alveolar component, but given the lower instilled volume, it is considered to be less invasive compared to BAL. Mini-BAL is poorly standardized. It has been reported as the instillation of a variable volume of saline solution (between 20 and 60 mL) using either the bronchoscope or a blind catheter advanced into a distal airway (47, 48). As for bronchial wash, this technique is suitable for microbiologic studies, but not for studying the alveolar component. Given the variability of the techniques used in different studies, it is difficult to evaluate the diagnostic accuracy of these techniques, but they are all reported to have a good safety profile.

In COVID-19 intubated patients, mini-BAL has been described in at least two studies. Vanbellinghen et al. retrospectively compared the prevalence of aspergillosis in COVID-19 diagnosed using mini-BAL (20 mL of saline instilled through a blind catheter) to that of BAL (47). The authors performed mini-BAL in 40 cases, BAL in 20, and both in 16 cases, showing a good agreement between the two methods and a similar prevalence of overall positive *Aspergillus* results using PCR and/or galactomannan and/or culture (16.7% BAL and 21.4% for mini-BAL) (47). Torrego et al. performed mini-BAL in 63 severe COVID-19 patients (all intubated, PaO2/FiO2 111, IQR 103–125), instilling 60 mL of saline with a wedged scope according to the radiological features (48). One-third were performed in the prone position. They had 28.6% (18/63) of positive microbiology results, with a profile of pathogens similar to what was observed in a retrospective pre-COVID-19 cohort of patients seen at their center (*Pseudomonas aeruginosa n* = 7, *Staphylococcus aureus n* = 2, *Klebsiella aerogenes n* = 2, *Enterobacter cloacae n* = 2, *Enterococcus faecalis n* = 2, *Escherichia coli n* = 1, *Streptococcus anginosus n* = 1, or *Prevotella melaninogenica n* = 1) (48). In Table 1, we reported a higher prevalence of coinfections detected by BAL in critically ill patients (21–54% critically ill, compared to 2–37% non critically ill). The comparison that Torrego made with their historical cohort suggests that this is related to the peculiar profile of respiratory infections observed in ICU patients.

To the best of our knowledge, only Mondoni et al. published a retrospective study that attempted to compare BAL to bronchial washing (BW) in suspected COVID-19 non-critically ill patients. The authors reported an overall diagnostic yield for SARS-CoV-2 detection of 55% (43/78), 57% (35/61) with BAL, and 47% (8/17) with BW, and the statistical difference was not reached (*p* = 0.45).

All these bronchoscopy procedures are similarly well-tolerated, but safety studies designed to compare these different methods are lacking.

We believe that the paucity of data does not allow us to clearly define whether one technique is superior to another. Many experienced centers prefer the use of BAL, which is the best standardized method to collect alveolar samples. BAL is superior to any other technique for collecting the alveolar cellularity and supernatant. However, particularly when the only aim is to detect SARS-CoV-2 or other infections, there is currently insufficient evidence to claim its superiority compared to other sampling methods (mini BAL or bronchial wash).

## BAL in COVID-19: cellularity, immunophenotype, and cytokine profile

BAL characteristics and cellularity can be extremely useful in clinical practice, helping to identify possible differential diagnoses and to guide the diagnostic and therapeutic choice of clinicians. BAL and lung cryobiopsy represent unique specimens to investigate the excessive inflammatory pulmonary response to SARS-CoV-2, which represents a major cause of disease severity and death (49, 50). Doglioni et al. elegantly described the histological and immunohistochemical features observed in the early-phase COVID-19 in cryobiopsies performed in non-intubated patients, with perivascular CD4-T-cell infiltration, capillary and venular changes, florid alveolar type II cells hyperplasia, and no hyaline membranes (50). The T-cell perivascular infiltrate was CD 4 positive but negative for functional activation markers (T-BET, FOXP3, CD 25, and CD 30). Few interstitial PD1 + and TCF 1+ T CD8+ lymphocytes were detected. NK cells (CD 56+) and B-cells (CD 20 +) were rare or absent (50). BAL studies can provide precious data on the cellular and molecular components from the distal lung that nicely integrate histology findings. Compared to lung biopsy, BAL is much more easily performed; therefore, a considerable number of recent studies have used BAL to evaluate the alveolar cellular profiles that could correlate with clinically meaningful outcomes (e.g., disease severity and mortality) and that could help the understanding of COVID-19 pathogenesis. Dentone et al. described the BAL characteristics and cellularity of 64 COVID-19 patients admitted during March and April 2020 to the Intensive Care Unit (ICU) of Genoa Hospital; 34.4% of cases had coinfections detected by BAL (*Candida*, *Psedumononas*, *Enterobacter aerogens*, *Staphylococcus aureus*, and *Klebsiella Pneumoniae*) (51). BAL samples from individual patients were taken, and analysed their total cellularity, subpopulations, and T lymphocyte activation as HLA-DR expression (51). The median cellularity was 68 × 10^3^/mL (IQR 20–145). The majority of cells in BAL were neutrophils (70%, IQR 37.5–90.5), followed by macrophages (27% IQR 7–49). Eosinophils were less than 1% (IQR 0.9–3). Lymphocytes were a minority, 1%, with CD3+ 92% (IQR 82–95). Among CD3+ T lymphocytes, 52% were CD8+ (IQR 39.5–62.7), with a T CD4+/CD8+ ratio of 0.6 (IQR 0.4–1.2); and 20% were HLA-DR+ (IQR 13–32). In multivariate analysis, only the percentage of macrophages in the BALF at the time of ICU entry correlated with higher mortality (OR 1.336, 95%CI 1.014–1.759, *p* = 0.039). The duration of mechanical ventilation was correlated with percentage of TCD8+ in BALF (*r* = − 0.410, *p* = 0.008), TCD4+/CD8+ ratio (*r* = 0.425, *p* = 0.006), and total lymphocytes TCD3+ (*r* = 0.359, *p* = 0.013) in BALF, respectively. The authors speculate that the lack of lymphocytes in the BALF in patients admitted to the ICU could partly explain a reduced antiviral response. The reason for this depression of lymphocytes could be related to both direct virus damage to the lymphocyte and cytokine storm-induced damage (51). Pandolfi et al. also confirmed that innate immunity is extensively activated, that the BALFs of 33 adults admitted to the ICU reported a marked increase in neutrophils (1.24 × 10^5^ mL, 0.85–2.07) and reduced numbers of lymphocytes (0.97 × 10^5^ mL, 0.024–0.34) and macrophages (0.43 × 10^5^ mL, 0.34–1.62), with viral particles inside mononuclear cells (seen by electron transmission microscopy and immunostaining) (52). The majority of BAL showed coinfections (26/28). The burden of pro-inflammatory cytokines was associated with clinical outcome, and IL-6 and IL-8 were significantly higher in ICU patients than those in the Internal Medicine Ward (IL6 *p* < 0.01, IL8 *p* < 0.0001) and also in those who did not survive (IL6 *p* < 0.05, IL8 *p* = 0.05 vs. survivors) (52). Another interesting pathogenetic mechanism of acute COVID-19 is represented by NETosis, a form of neutrophil death leading to the formation of neutrophils extracellular traps (NETs) of extracellular chromatin and assembling proteins, including antiviral proteins (53). This form of host defense is related to the well-known cytokine storm observed in acute COVID-19. NETs increase in BAL of Adult Respiratory Distress Syndrome (ARDS) cases has been described in the past (54). Recently, Martens et al. reported an increase of NET biomarkers in the BAL of severe COVID-19, thus suggesting a possible potential new therapeutic target in severe COVID-19. Meloni et al. investigated 33 BAL of COVID-19 patients, finding a high burden of both human neutrophil elastase (HNE) and α1-antitrypsin that, despite its ability to bind histones, was not able to block HNE activity and prevent NET formation (55). A recent study by Reynolds and co-workers showed that inflammatory immune dysregulation of the lower airways during severe viral pneumonia (both severe influenza and SARS-CoV-2 were included) is distinct from that of non-severe illness, with an influx of non-classical monocytes, activated T cells, and plasmablasts B cells. BAL cytokines were elevated in severe cases but not in moderate patients. The largest elevations were observed in IL-6, IP-10, MP-1, and IL-8 (56). In contrast to previous reports, Gelarden et al. reported lymphocytosis (i.e., >15%) in 74.7% of cases (62/83) intubated for severe COVID-19 with a high prevalence of atypical lymphocytes in BAL (72.3%, 60/83) (57). BAL lymphocytes, including plasmacytoid and plasmablastic cells, were composed predominantly of T cells with a mixture of CD4+ and CD8+ cells. Both populations had increased expression of T-cell activation markers, suggesting important roles of helper and cytotoxic T-cells in the immune response to SARS-CoV-2 infection in the lung. BAL lymphocytosis was significantly associated with longer hospital stay (*p* < 0.05) and longer requirement for mechanical ventilation (*p* < 0.05), whereas the median atypical (activated) lymphocyte count was associated with shorter hospital stay (*p* < 0.05), shorter time on mechanical ventilation (*p* < 0.05), and improved survival (57). All these data should be interpreted with great caution because they are derived from small, retrospective, and monocentric studies with an evident heterogeneity between cohorts in terms of phenotypes, disease severity, duration of intubation, and presence of coinfections. Moreover, there is a critical lack of BAL data in non-intubated patients with less severe COVID-19, which limits our ability to understand disease pathogenesis in the early phase of the disease. In addition to those evident limits, the current body of evidence suggests that BAL cellular analysis is an invaluable tool to provide useful information for diagnostic and prognostic workup and potentially expand our understanding of COVID-19 pathogenesis.

## COVID-19 single cells studies in BAL

The majority of single-cell studies to date were performed on peripheral blood mononuclear cells (PBMC), a minority on NP swabs and BAL. Few studies have dissected the epithelial and immune profiles of BAL derived from severe COVID-19 patients at a single-cell level. Wauters et al. revealed infected lung epithelial cells, a significant proportion of neutrophils and macrophages involved in viral clearance (58). They performed single-cell deep-immune profiling BAL from 5 patients with mild COVID-19 and 26 with critical COVID-19 (compared to non-COVID-19 pneumonia and normal lung), showing divergent immunologic profiles. In mild COVID-19, CD8+ resident-memory (TRM) and CD4+ T-helper-17 (TH17) cells undergo active expansion with good effector functions, while in critical cases, they remain more naïve. Vice versa, CD4+ T-cells with T-helper-1 characteristics (TH1-like) and CD8+ T-cells expressing exhaustion markers (TEX-like) are enriched halfway through their trajectories in mild COVID-19, where they also exhibit good effector functions, while in critical COVID-19, they show evidence of inflammation-associated stress. Monocyte-to-macrophage trajectories show that chronic hyperinflammatory monocytes are enriched in critical COVID-19, while alveolar macrophages, otherwise characterized by anti-inflammatory and antigen-presenting characteristics, are depleted. Moreover, in critical COVID-19, monocytes contribute to an ATP-purinergic signaling-inflammasome footprint that could enable COVID-19-associated fibrosis and worsen disease severity (58). Liao et al. evaluated BAL from 3 moderate and 6 severe COVID-19 and found abundant pro-inflammatory monocyte-derived macrophages in patients with severe COVID-19, whereas highly clonally expanded CD8+ T cells characterized average COVID-19 cases (59). Patients with severe/critical infection had much higher levels of inflammatory cytokines, particularly interleukin (IL)-8, IL-6, and IL-1β, expressed by macrophages that in severe patients may contribute to local inflammation by recruiting monocytic cells and neutrophils through CCR1 and CXCR2, while in moderate cases can produce more T cell attracting chemokines through CXCR3 and CXCR6 (59). He et al. performed single-cell RNA sequencing (sc-RNA-seq) in the leukocytes and epithelial cells of 3 SARS-CoV-2-induced ARDS (60). They detected 23 cells with viral mRNA reads but a minimal number of expressed genes, thus indicating that SARS-CoV-2 suppresses host gene expression. These cells were identified as monocytes/neutrophils and club cells. Compared to healthy controls, club cells showed a significantly elevated mucins gene expression (MUC5AC, MUC5B, MUC4, MUC16, and MUC20). The mucin secretion seems stimulated through the innate immune regulators IL-1β and TNF-α (found 6 transcription factors were involved in IL-1β and TNF-α-induced MUC5B promoter activation). Four critical surfactant proteins (SPs), SP-A, SP-B, SP-C, and SP-D, known to maintain the structural integrity of alveoli, were downregulated in COVID-19 disease, and the level of NKX2-1, the transcription factor required for surfactant synthesis, was also reduced, thus indicating the loss of alveoli integrity and the possible pathogenesis of ARDS in COVID-19 (60). The transcriptomic signature of major regulators of innate immunity (monocytes, neutrophils, and macrophages) in severe COVID-19 indicates different immune profiles among COVID-19 patients: Liao et al. showed abundant macrophages expressing FCN1 in BAL of COVID-19 patients, whereas He et al. noticed only a minor increase in FCN1+ macrophages, with a significant decrease in FCN1+ monocytes/neutrophils (59, 60). By analyzing scRNA-seq data of BAL from 6 severe COVID-19, 3 recovered COVID-19 with mild symptoms and 10 heathy controls, Chen et al. showed high expression of SARS-CoV-2 receptor ACE2 and TMPRSS2 in club and ciliated cells of patients (61). In severe COVID-19 patients, high neutrophils with excessive expression of cytokines were noted and the dysregulated cytokines/receptors interplay among lung epithelial cells and immune cells correlated with disease severity (ANXA1/FPR2 and TNFSF13/TNFRSF1A interactions between club and macrophage or neutrophils, CXCL2/DPP4 interaction between club and T/NK cells, and ANXA1, C3, CXCL2, SAA1, and TNFSF13 expressions in lung epithelial cells) (61). Recently, Karmaus et al. performed a meta-analysis of BAL scRNA-seq data noting significant reductions of inferred NKX2-1 and NR4A1 activities in alveolar epithelial type II (AT-II) cells and showing changes in inferred AT-II cell metabolic activity, increased transitional cells, and a previously undescribed AT-I state characterized by the induction of genes of the epidermal differentiation complex, including the cornified envelope protein SPRR3 (small proline-rich protein 3), upregulation of KRT (keratin) genes, inferred mitochondrial dysfunction, and cell death signatures (apoptosis and ferroptosis) (62).

In conclusion, scRNA-seq studies can reveal information of critical importance in the understanding of COVID-19 pathogenesis. However, current data on BAL are limited, mostly derived from small sample size studies, and there are large difficulties in validating most conclusions across datasets, possibly due to inconsistent mapping between different disease stages and different protocols used. Therefore, conclusions from these early scRNA-seq studies of COVID-19 patients may not always be robust and need to be validated before being fully relied upon (63).

## Bronchoscopy complications

BAL is reported to be safe, but a transient drop in oxygen saturation is occasionally reported in the more severe patients. No major adverse events were reported to date and no deaths were recorded. The most frequent adverse events, described in a minority of patients, were transient hypoxemia and fever. Mondoni et al. reported complications related to bronchoscopy in 5/109 (4.5%) patients. Fever was recorded after BAL in 2/109 (1.8%); 3/109 (2.7%) patients with a known mild respiratory failure had a transient worsening of their gas exchange after bronchoscopy performed during oxygen supplementation. When bronchoscopy was performed in patients who required non-invasive mechanical ventilation (NIV), severe hypoxia and subsequent intubation has been reported in 6 patients (18).

In COVID-19, BAL is reported as a safe and feasible procedure in all studies, with a safety profile that is similar to what was previously reported in non-COVID-19 patients. The risk–benefit profile should be carefully evaluated in severe patients in NIV because of the possible risk of hypoxemia leading to intubation. The small numbers and the wide heterogeneity of studies prevent us from drawing any firm conclusion on possible differences in terms of safety and diagnostic accuracy between BAL and other sampling techniques, such as mini-BAL and bronchial washes. Future prospective trials are needed to address the safety and accuracy of these methods.

## Healthcare workers safety

In the published studies, all bronchoscopies were performed in accordance with current guidelines using appropriate personal protection equipment (PPE), including gown, face shield, eye protector, shoe cover, double gloves, and filtering masks (FFP2/FFP3) (18). Negative pressure rooms and disposable bronchoscopes were not universally available, although they were used in the majority of centers (negative pressure rooms in 57%, 4/7 studies; disposable scopes in 67%, 6/9 studies) (18). Among all published studies (646 patients, 1,034 bronchoscopies), only Torrego et al. reported one infection in a bronchoscopist (18, 48). Based on current evidence, we can conclude that, if performed with appropriate PPE, bronchoscopy and BAL can be safely performed with minimal risk of infection for healthcare workers.

## Conclusion

**Figure 1:**
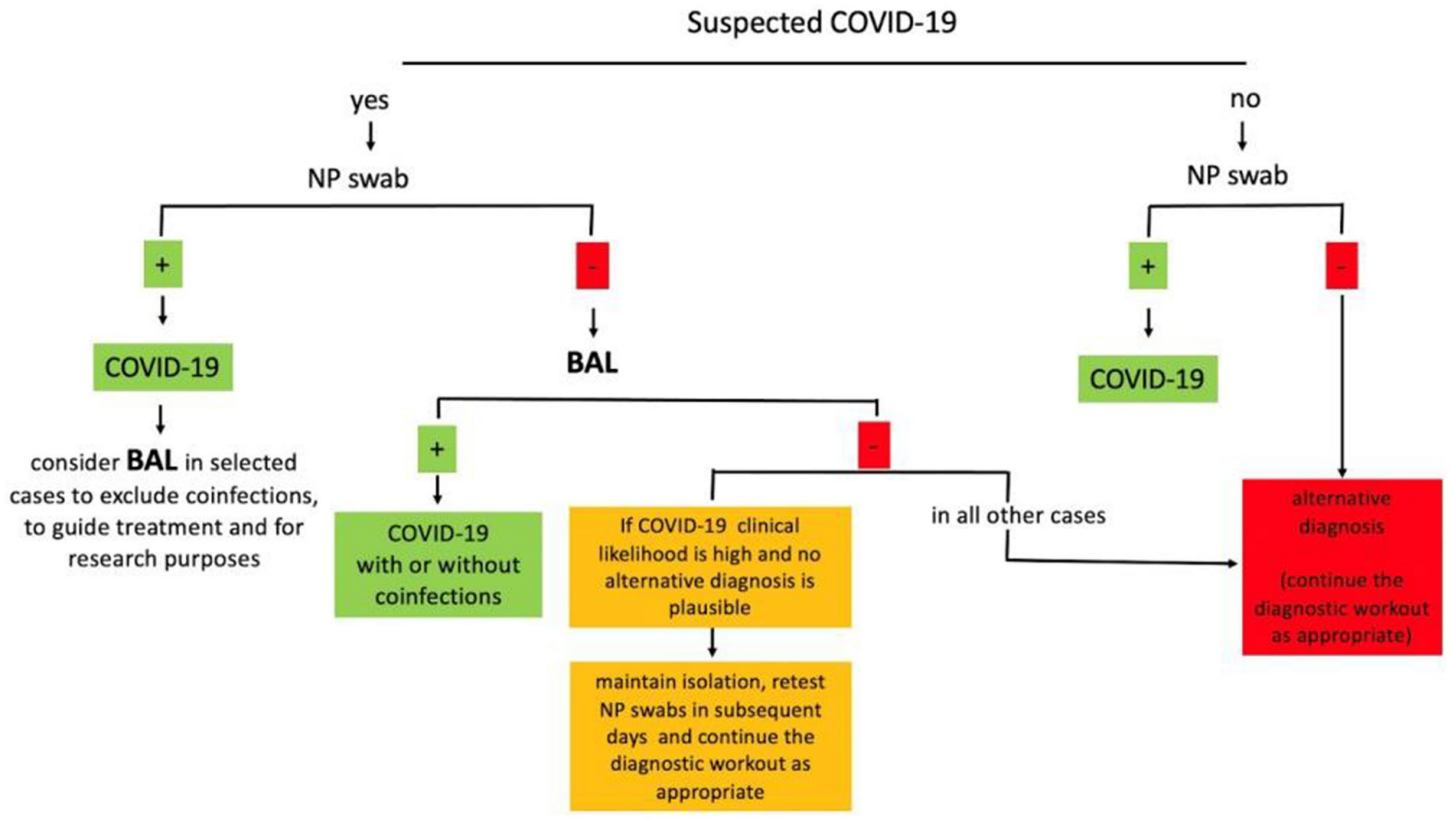
Simplified diagnostic algorithm for the use of BAL in suspected COVID-19. BAL, bronchoalveolar lavage; NP, nasopharyngeal.

BAL has been widely used during the SARS-CoV-2 pandemic for both clinical and research purposes. In clinical practice, BAL can change management decisions in up to two-thirds of patients, confirming a suspected SARS-CoV-2 infection when the NP swab is negative, detecting other infections, or supporting the alternative diagnosis. Although studies have wide variability, pooled estimates of 11% positive cases suggest that BAL can be used to confirm suspected SARS-CoV-2 infections when negative NP swabs are negative (19). The prevalence of false negative BAL for SARS-CoV-2 detection cannot be accurately drawn from current studies but seems to be very low (<2%) (25). In both critically ill and non-critically ill patients, BAL detects coinfections in a significant proportion of patients. BAL can help clinicians in difficult differential diagnoses, including acute exacerbations of interstitial lung diseases (ILDs), connective tissue-related ILDs, hypersensitivity pneumonitis, and cryptogenic organizing pneumonia. BAL analysis are used to guide steroid and immunosuppressive treatments and narrow or discontinue antibiotic treatment, reducing the use of unnecessary broad antibiotics. Moreover, cellular analysis and novel multi-omics techniques on BAL are of critical importance for the understanding of the microenvironment and interaction between epithelial cells and immunity, revealing novel potential prognostic and therapeutic targets. The BAL technique has been described as safe for both patients and healthcare workers in more than a thousand procedures reported to date in the literature. Based on these preliminary studies, we recognize that BAL is a feasible procedure in COVID-19 known or suspected cases, useful to properly guide patient management, and with great potential for research. Based on the evidence summarized here, we propose a simplified diagnostic algorithm in which BAL can be used in suspected COVID-19 cases when the NP swab is negative and in COVID-19 cases to guide antimicrobial and steroid treatment when a coinfection is suspected (Figure 1). The proposed algorithm can be applied at hospital admission, when a timely diagnosis of COVID-19 is essential to properly allocate patients in the dedicated areas. Patients at higher risk for complications or poor outcomes should be promptly considered for a BAL evaluation to guide an early and appropriate antiviral and/or antibiotic treatment. In future pandemics, novel clinical and radiological quantitative analysis and scoring systems, along with emerging BAL biomarkers, will hopefully support clinicians in making management decisions. We acknowledge that this algorithm reflects the clinical practice only in selected centers properly equipped and experienced in the use of BAL and that further large prospective studies are needed to corroborate current knowledge before BAL can be widely recommended.

Even if the pandemic is resolved now, finding adequate strategies to be prepared for possible future pandemics remains of great importance.

## Sharable abstract

COVID-19 diagnosis can be challenging. BAL has shown to be feasible and useful in the diagnosis and management of both COVID-19 and its complications. This review summarises the evidences on the clinical and research utility of BAL in COVID-19 patients.

## Plain language summary

COVID-19 diagnosis and management can be challenging. BAL has shown to be feasible and useful in the diagnosis of COVID-19 and in the detection of coinfections, helping clinicians to guide appropriate treatment. Moreover, BAL is a potent research tool that is providing novel insights in the understanding of COVID-19 pathophysiology.

## Author contributions

ST: Writing – original draft, Writing – review & editing. LC: Writing – original draft, Writing – review & editing. VL: Writing – review & editing. LGo: Writing – review & editing. MT: Writing – review & editing. LGi: Writing – review & editing. FL: Writing – review & editing. VP: Writing – review & editing. CR: Writing – review & editing. AT: Writing – review & editing. FM: Writing – review & editing. RoL: Writing – review & editing. FA: Writing – review & editing. LM: Writing – review & editing. GR: Writing – review & editing. SP: Writing – review & editing. OP: Writing – review & editing. GC: Writing – review & editing. AC: Writing – review & editing. LR: Writing – review & editing. AB: Writing – review & editing. MS: Writing – review & editing. MM: Writing – review & editing. SG: Writing – review & editing. CC: Writing – review & editing. MC: Writing – review & editing. AP: Writing – review & editing. MH: Writing – review & editing. BE: Writing – review & editing. ReL: Writing – review & editing. J-lC: Writing – review & editing. CN: Writing – review & editing. JG: Writing – review & editing.
